# Daises and Buttercups

**Published:** 1889-07-27

**Authors:** 


### Daisies and Buttercups.

The Daisy and Buttercup Fund is intended to send poor children to the country for a holiday, a preference being given to those children who are employed in theatres. So it is perfectly fitting that an addition to its funds should be provided by a play in which children should be the chief players. And if we had wanted to convince Mrs. Fawcett, Mr. Smith, and the other people who object to the employment of children in theatres, that there is something to be said on the other side, we should have invited them to go to Lowther Lodge on Thursday, the 18th, and see Mr. Ben Greet's company of "Woodland Players" in the "Midsummer Night's Dream." So performed, it was not a stage play in the conventional sense of the term; at least, the greensward was the stage and the hawthorn brake the tiring-room, as Peter Quince, bellowsmender and stage manager, pointed out to his "mechanical" company. The moonlight was artificial; all else was real. And for this reason, perhaps, the "Midsummer Night's Dream," which has not been successful in the theatre within the memory of living man, was a perfect success in the garden of Lowther Lodge. The complicated and changeable loves of Helena and Hermia and their swains did, indeed, seem rather artificial—that being, in fact, the most conventional part of the play; but the amateur players and the fairies seemed perfectly real. Bottom, the weaver, the aspiring *jeune premier* who wanted to play all the leading parts in the play—a personage not unknown to modern life—was perfectly given by Mr. Ben Greet; and his asinine gambols when, after his "translation" by Puck, all Titania's caresses cannot keep him from "kicking up his heels," were delightful to witness. Miss Mabel Dent, who played the "tricksy spirit," is a born Puck. The quaintness, the humour, the complete appreciation of the spirit of the part which this tiny maid displayed—she cannot be more than seven or eight years old—showed not only wonderful natural intelligence, but careful and kindly training, for you can never frighten a child into cleverness. All the fairies were children, and their small size, combined with their long gauzy robes and unfettered grace of motion—so different from the dress and poses of the ordinary ballet-girl—had a charming effect. The whole performance was as charming as it was unique; and the only regret one felt was that in England such performances can never become more common.

